# Bioclimatic Zoning for Sheep Farming through Geostatistical Modeling in the State of Pernambuco, Brazil

**DOI:** 10.3390/ani13061124

**Published:** 2023-03-22

**Authors:** Gabriel Thales Barboza Marinho, Héliton Pandorfi, Marcos Vinícius da Silva, Abelardo Antônio de Assunção Montenegro, Lizandra de Barros de Sousa, Raquel Desenzi, Jhon Lennon Bezerra da Silva, José Francisco de Oliveira-Júnior, Márcio Mesquita, Gledson Luiz Pontes de Almeida, Cristiane Guiselini, Alexandre Maniçoba da Rosa Ferraz Jardim, Thieres George Freire da Silva

**Affiliations:** 1 Department of Agricultural Engineering, Federal Rural University of Pernambuco, Recife 52171-900, Brazil; 2 Department of Veterinary Medicine, Federal Rural University of Pernambuco, Recife 52171-900, Brazil; 3 National Institute of the Semiarid, Center for Information Management and Popularization of Science, Campina Grande 58434-700, Brazil; 4 Institute of Atmospheric Sciences, Federal University of Alagoas, Maceió 57072-260, Brazil; 5 Department of Agronomy, Federal University of Goiás, Goiânia 74690-900, Brazil

**Keywords:** spatial analysis, thermal comfort index, biometeorology, GIS, Northeast of Brazil

## Abstract

**Simple Summary:**

Heat stress (HS) is a complex phenomenon that triggers multiple animal response mechanisms that negatively impact livestock welfare and production. Thermal comfort is therefore an important subject for limiting performance loss and other adverse effects of heat stress on animal physiology in different production systems; furthermore, it is becoming increasingly important in light of recent climate change scenarios. The purpose of this study was to point out different areas of Pernambuco state that are likely to be best suited to different sheep breeds. The study identified two dairy breeds (East Friesian and Lacaune) that have good potential to be farmed in specific areas of Pernambuco state. The thermal comfort indices presented in Pernambuco were favorable for the main meat-producing breeds.

**Abstract:**

The Intergovernmental Panel on Climate Change (IPCC) has pointed out the high vulnerability of developing countries to climate change, which is expected to impact food and income security. Sheep farming is one of the main animal productions among the families located in the most vulnerable regions of the semiarid region of Pernambuco state, a Brazilian territory known for its high temperatures, low relative humidity, and high net solar radiation. Therefore, the objective of this study was to identify different regions of Pernambuco that may be more suitable for different breeds of sheep, based on non-parametric statistics and kriging maps of the temperature and humidity index (THI). THI values were determined based on mean annual temperature and wind speed extracted from the TerraClimate remote sensing database. Pernambuco state presented THI values ranging from 66 to 79, with the hair breeds having a high potential for exploitation in almost all territories, including the main meat-producing breeds. The East Friesian breed, a high milk producer, would be well suited to the Agreste mesoregion, a territory that, like the Pajeú and Moxotó microregions, also proved favorable for the introduction of three wool breeds (Suffolk, Poll Dorset, and Texel) known as major meat producers. The kriging maps of the THI values successfully allowed the identification of strategic development regions of Pernambuco state with high potential for sheep breeding.

## 1. Introduction

Heat stress is the result of a combination of climatic variables, including wind speed, high temperature, air humidity, and high solar radiation, that adversely impact animal welfare and productivity [1]. An animal is considered stressed when it needs to alter its physiology and behavior to adapt to adverse environmental and management conditions [2]. Under current climate conditions, herds are already in this state of stress, with a marked increase compared to the period of 1981–2010 [3].

Several indices have been used to assess the level of stress in livestock caused by climatic conditions, with the temperature and humidity index (THI) being popular [4,5,6,7,8]. Environmental conditions such as high temperature, insolation, low relative humidity, and insufficient rainfall are prominent features of Brazil’s semiarid regions [9,10]. Extreme temperatures, such as 38 °C, can be reached in this region during the hottest season, which means animals are subject to chronic heat stress [11,12]. These excessive heat conditions can severely impact the welfare and behavior of sheep (*Ovis aries*), affecting their productivity, since heat stress is a limiting factor for these animals [13,14].

According to the Brazilian Institute of Geography and Statistics—IBGE [15], Pernambuco state jumped from the fifth largest national producer of sheep in 2006, to the third position in 2015 and reaching the second position in 2020, with Bahia state being the largest national producer with 4.25 million sheep units. With about 3.43 million animals, the sheep herd in Pernambuco is the second largest in the state, and of this total, about 3.36 million are located in the mesoregions of São Francisco (45.05%), Sertão (36.98%), and Agreste (16.24%) [15].

In the Northeast of Brazil (NEB), meat and leather have always been the main productive chains, but Hermuche et al. [16] highlighted that the vast majority of sheep production in Brazil comes from very low-tech farms in marginal areas, using traditional production systems. In a harsh environment, with extensive production systems, as in the semiarid part of the NEB, the most abundant breeds are the smallest sheep with hair, such as the Morada Nova, Somalis Brasileira, and Rabo Largo [17].

Because of the differences in sheep production systems in the NEB semiarid region [18], it is difficult to know precisely the number of flocks of each breed present in the region. McManus et al. [19] showed that hair sheep are more often raised in smaller flocks in more stressful regions of the Northeast and Midwest. In addition, the same study showed the prevalence of the breeds Morada Nova, Rabo Largo, and Cariri in Pernambuco state, east of the NEB (ENEB). However, the breeding of sheep in the Brazilian semiarid region is characterized as traditional family farming, and it is very common to use the Santa Inês breed to increase the meat and milk production of the locally adapted breeds [18,20].

In NEB sheep farming, in general, native animals predominate, without a defined racial pattern, showing excellent adaptability to the heat conditions of the region [21]. Santa Inês and Suffolk rams were brought to the Northeast of Brazil to improve the body composition of the breed, and successive selections for wool shortages resulted in an increase in the proportion of black and brown Santa Inês [22], which became dominant, causing lighter-colored animals to be less well regarded by producers, as they tend to show weaker body development [23].

Bioclimatic zoning, by monitoring climatic conditions, allows the prediction of areas with a high probability of heat stress occurrence. Zoning helps in making decisions regarding environmental management to minimize heat stress, such as shading for animals not confined in the hottest hours of the day. In situations of heat discomfort, ruminants choose to reduce the time dedicated to social interactions of reproductive nature, consequently increasing their leisure time, which explains the inverse relationship between heat stress indicators and animal activity [24].

Under heat stress, physiological changes assist ruminants to cope with the challenges imposed [25]. Polli et al. [26] reported that in the southern region of Brazil, lambs exposed to a mean temperature of 23.4 °C experienced heat stress for 27.7% more of the total confinement time compared to lambs at a mean temperature of 14.9 °C. It is also important to note that the thermoregulatory mechanisms required to reverse adverse effects of heat stress eventually become detrimental to the overall performance of sheep [27,28,29].

Geostatistics, through kriging maps of meteorological variables or indexes, is often used to perform precise zoning for animals and crops, also allowing the prediction of areas with a high probability of heat stress occurrence [30,31,32,33,34], which can be measured through a series of physiological and behavioral variables. Mendes et al. [21] used geostatistics to perform a bioclimatic zoning for the Dorper breed in Pernambuco state. However, there are no data or scientific studies involving the other breeds that are relevant for the economic development and food security of the population present mainly in the semiarid region of the state. Thus, to fill the knowledge gaps in this existing area, this study aimed to identify different regions of Pernambuco state that are more suitable for several sheep breeds based on non-parametric statistics and kriging maps of annual THI values. Such maps are strategic for development plans for the region in the near future, enhancing adaptation to climate change.

## 2. Materials and Methods

### 2.1. Characterization of the Study Area

**Figure 1:**
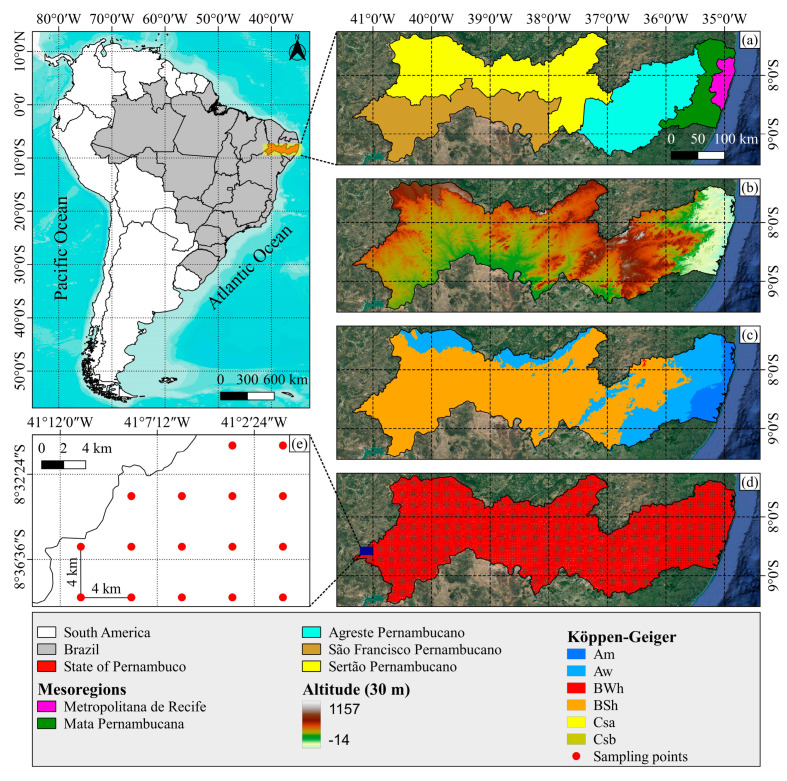
(**a**) Location of the study area. (**b**) Digital elevation in meters, based on Shuttle Radar Topography Mission (SRTM) data. (**c**) Climatic zones according to the Köppen–Geiger climate classification. (**d**) Sampling grid obtained from TerraClimate. (**e**) Sampling points with spatial resolution of 4 km (1/24°).

This study was carried out in Pernambuco state—inserted in the NEB region, which covers an area of 98,312 km^2^, situated between the parallels of 7°15′45″ and 9°28′18″ S and the meridians of 34°48′35″ and 41°19′54″ W. According to the Köppen–Geiger climate classification, the region’s climate divides into the following six classes: Am (humid tropical climate); Aw (tropical savanna climate with a dry winter season); BWh (hot arid climate); BSh (hot semiarid tropical climate with a defined dry season); Csa (hot summer Mediterranean climate); and Csb (cool summer Mediterranean climate) (Figure 1) [35].

Throughout most of the year, the semiarid region of Pernambuco presents high temperatures, with an average annual minimum and maximum of 24 and 29 °C, respectively, reaching 35 to 38 °C during the hottest hours. The intense solar radiation in the region accumulates an annual energy of 2.2 MWh·m^−2^ with the average daily solar irradiation higher than 5.0 kWh·m^−2^·day^−1^ [36].

### 2.2. Climatological and Geospatial Data

The data used in this study came from the TerraClimate database, which comprises water balance and climate data, with a monthly frequency and spatial resolution of approximately 4 km (1/24°) and time series from 1958 to the present time [37]. The TerraClimate dataset is divided into primary and secondary climate variables. The primary climate variables are maximum temperature, minimum temperature, vapor pressure, total precipitation, downwelling surface shortwave radiation, and wind speed. On the other hand, the secondary climate variables are reference evapotranspiration (ASCE Penman–Monteith standardized model), runoff, actual evapotranspiration, climatic water deficiency, soil moisture, equivalent water snow, Palmer Drought Severity Index (PDSI), and vapor pressure deficit.

For the present study, maximum and minimum air temperature (Tmax and Tmin, °C) and mean wind speed (Ws, m.s^−1^) were used, totaling 4653 observations (sampling points) (Figure 1) distributed throughout Pernambuco state. Data were obtained from the Climate Engine platform (https://climateengine.com/, accessed on 12 September 2022), the image processing platform from TerraClimate, as well as the point data georeferencing. Subsequently, the mean air temperature (Tair, °C) was obtained, and then the annual mean temperature and humidity index from 2010 to 2021 was estimated, as well as the fractional behavior of this index in the study region [38,39,40,41,42,43], according to Equation (1) established by [40]:

(1) $$\mathrm{THI}=(6.3952+0.08964\;\mathrm{Tair}+0.01018{\;\mathrm{Ws})}^{2}$$

### 2.3. Statistical Analysis

The spatiotemporal data were submitted to descriptive statistical analysis to obtain the mean, median, minimum, maximum, standard deviation (SD) and coefficients of variation (CV, %), and asymmetry and kurtosis. The percentage value of the CV was categorized as low (CV < 12%), medium (CV = 12–24%), or high (CV > 24%) [44]. Subsequently, the Kolmogorov–Smirnov (KS) normality test was also applied, using a significance level of *p* ≤ 0.01.

### 2.4. Geostatistical Analysis

Kriging is a regression method used to interpolate data that takes into account the spatial autocorrelation characteristics of regionalized variables, using a mean structure and a Gaussian stochastic process, assuming that points close together in space tend to have more similar values than points farther apart [45,46,47]. By taking into account the existence of spatial continuity, it allows data obtained by sampling certain points to be used for the estimation of points where the value of the variable is unknown.

To investigate the spatial structure of variation, geostatistical analysis based on classic semivariances was adopted, according to Equation (2), which estimates the degree of spatial dependence between the pairs of observations. The magnitude of the semivariance between two points depends on the distance between them, implying smaller semivariances for smaller distances and larger semivariances for larger distances [48,49]. The plot of semivariance as a function of distance to a point is called a semivariogram.

Semivariance calculation, based on Equation (2), semivariogram function model fitting, and cross-validation were performed using the geostatistical software ArcMap 10.5 from Environmental Systems Research Institute (ESRI).

(2) $$\gamma \left(h\right)=\frac{1}{2N(h)}\overset{N(h)}{\underset{i=1}{\sum }}[Z\left(X_{i}\right)-{\;Z(X}_{i}+h)]\text{²}\;$$

where γ(h) is the experimental semivariance estimator, obtained by the sampled values Z(X_i_), Z(X_i_ + h); N(h) is the number of measured value pairs separated by the vector or lag distance; h is the distance between sample pairs; and Z(X_i_) and Z(X_i_ + h) are the values of the i-th observation of the regionalized variable, collected at points X_i_ and X_i_ + h (i = 1, …, n), separated by the h vector.

The raw data were imported to ArcMap, and experimental semivariograms were calculated. Three variogram models (i.e., experimental, Gaussian, and spherical) were fitted to the experimental semivariogram [50].

Spherical Model:

(3) $$y\left(h\right){=\text{\{}C}_{0\;}+C\left[1.5\frac{h}{a}-\;0.5{\left(\frac{h}{a}\right)}^{3}\right],\;\mathrm{for}\;0\;\leq \;h\;\leq {\;a\;C}_{0\;}+C,\;\mathrm{for}\;h\;\text{>}\;a\;$$

Exponential Model:

(4) $$y\left(h\right){=C}_{0}+C\left[1\;-\;\exp\left(-\frac{3h}{a}\right)\right]\;\;\;\;\;\;\;\;\;\;$$

Gaussian Model:

(5) $$y\left(h\right){=C}_{0}+C\left[1\;-\;\exp\left(-\frac{{3h}^{2}}{a^{2}}\right)\right]\;\;\;\;\;\;\;\;\;\;$$

where γ(h) is the experimental semi-variance estimator; C_0_ + C is the sill; C_0_ is the nugget effect; C is the variance dispersion; h is the distance between sample pairs; and *a* is the range (m).

As an alternative method for evaluating the model’s accuracy, deviations in the estimates from the measured data were compared by cross-validation [50,51]. This comparison of performance between the models was carried out using the following statistics: mean absolute error (MAE), mean error (ME), mean square error (MSE), average standardized error (ASE), root mean square error (RMSE), and root mean square standardized error (RMSSE). The five error statistics of predictions were applied to the cross-validation analysis. The equations are as follows [52]:

(6) $$\mathrm{ME}=\frac{1}{N}\overset{N}{\underset{i=1}{\sum }}\left[Z\left(x_{i}\right)-\hat{\;Z}{(x}_{i})\right]\;\;\;\;\;\;\;\;\;\;$$

(7) $$\mathrm{MSE}=\frac{1}{N}\overset{N}{\underset{i=1}{\sum }}\left[\frac{Z\left(x_{i}\right)-\hat{\;Z}\left(x_{i}\right)}{\sigma_{i}}\right]\;\;\;\;\;\;\;\;\;\;$$

(8) $$\mathrm{ASE}=\sqrt{\frac{1}{N}\overset{N}{\underset{i=1}{\sum }}{(\sigma }_{i})\;}$$

(9) $$\mathrm{RMSE}=\sqrt{\frac{1}{N}\overset{N}{\underset{i=1}{\sum }}\left[Z\left(x_{i}\right)-\hat{\;Z}{(x}_{i})\right]\text{²}}\;\;\;\;\;\;\;\;\;\;$$

(10) $$\mathrm{RMSSE}=\sqrt{\frac{1}{N}\overset{N}{\underset{i=1}{\sum }}\left\{\frac{Z\left(x_{i}\right)-\hat{\;Z}{(x}_{i})}{{(\sigma }_{i})}\right\}}\text{²}\;$$

where $\hat{Z}$($x_{i}$) is the predicted value, Z($x_{i}$) is the observed value, N is the number of values, and $\sigma_{i}$ is the standard error for location $x_{i}$.

The degree of spatial dependence (DSD), when less than 25%, was considered strong. Between 25 and 75%, it was considered moderate, and once greater than 75% it was considered weak [53].

## 3. Results and Discussion

### 3.1. Boxplot Analysis

**Figure 2:**
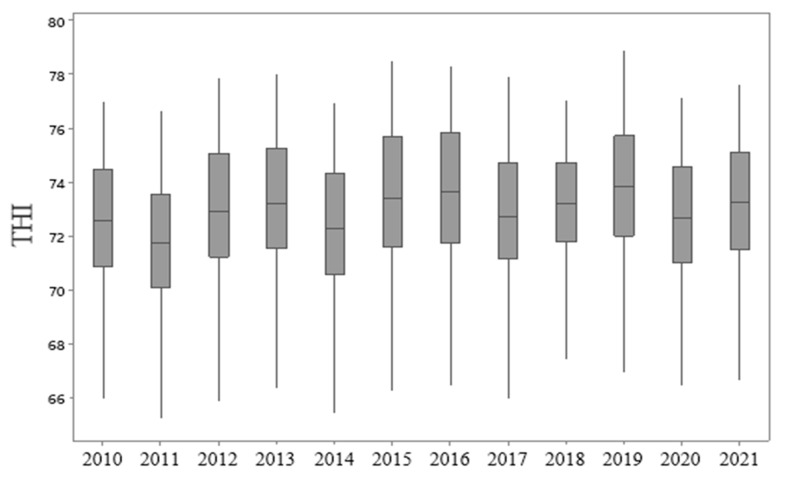
Boxplot of the annual THI of the meteorological stations over the years.

To identify outliers and some statistical properties, a boxplot analysis was performed in the 12-year series THI data used in this study, constituting the summary of 5 numbers (Figure 2), that is, the minimum and maximum values and 3 percentiles (median and interquartile range).

The resultant boxplots show that there is a significant pattern found in the dispersion of the index measured in this study. The data were homogeneous [54], and no outlier was found. The median was similar for all values, without a large discrepancy between the years, as can also be seen in Table 1. The result of the boxplot analysis indicates little asymmetry in the data studied [55], also shown by the position of the median line. The amplitude was similar for all the years studied, except for 2018, a year that was characterized by a great drought in the NEB [56,57], which showed a smaller amplitude when compared to the other years studied.

### 3.2. Descriptive and Geostatistical Analysis of THI

Table 1 presents descriptive statistics of annual THI values for the studied years, based on a general average. The CV was considered low (<24%) for all the years evaluated [44], indicating a high homogeneity of the data. As the mean and median values were similar, a behavior verified for all years evaluated in the present study (Table 1), the data were assumed as presenting normality, in accordance with Silva et al. [43,58].

The spherical, Gaussian, and exponential geostatistical models were tested individually for each year (Table 2).

The criteria for cross-validation were as follows: ME and MSE indicate the degree of bias in the model prediction and should be approximately zero; RMSE and ASE indicate the precision of prediction and their values should be as small as possible. RMSSE compares the error variance with the kriging variance and should be approximately 1. If RMSE is equal to ASE, then all errors are small. If ASE > RMSE or RMSSE, the model-predicted values are larger than the actual values [51,52,59].

**Figure 3:**
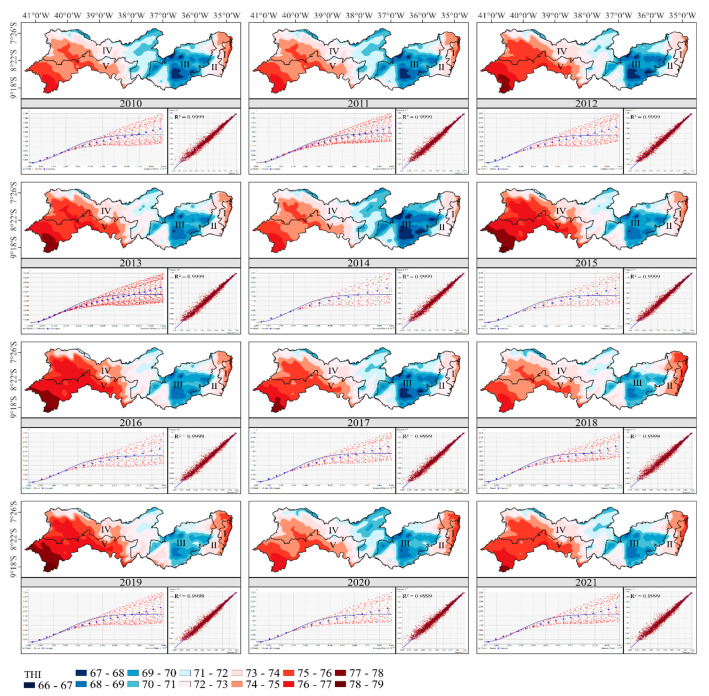
Kriging maps of THI, experimental semivariograms, and cross-validation for the years 2011 to 2021 in the Metropolitana, Zona da Mata, Agreste, Sertão, and São Francisco mesoregions of Pernambuco state, Brazil.

The criteria that the RMSSE should be close to 1 and that the RMSE and ASE values should be close to each other led to the Gaussian model being chosen and then being adopted for the kriging maps (Figure 3). In Table 2, we can see that for all years the Gaussian model presented RMSSE greater than 0.91, while for the other models the value was between 0.52 and 0.72. The determination coefficient (R²) showed an adequate fit, with values greater than 0.9, for all the years evaluated, as shown in Figure 3. The parameters (i.e., nugget effect, sill, and range) as well as the degree of spatial dependence of the geostatistical model used can be seen in Table 3.

### 3.3. THI Kriging Maps

Using the validated and established semivariogram models, maps of the spatiotemporal distribution of annual THI for Pernambuco were processed (Figure 3) by kriging. To facilitate the bioclimatic zoning, the map of Pernambuco state was divided according to the physiographic zones of its own mesoregions: I—Metropolitana; II—Zona da Mata; III—Agreste; IV—Sertão; and V—São Francisco.

The maximum variation in THI across the state ranged from 66 to 79, where the higher the value, the more stressful the environment [60]. However, the threshold value, at which point the animal starts to be stressed, depends on the species and even the breed [19,61,62]. The São Francisco mesoregion presented the highest THI values in the state, while Agreste was characterized by the lowest values. The Metropolitana mesoregion and a large part of Zona da Mata are coastal regions; therefore, they are subject to high temperatures associated with high relative humidity [57,58,63], a characteristic that may have contributed to the high THI values in these regions, especially in the eastern part of the Metropolitana area. These results corroborate with the study carried out by Mendes et al. [21], which identified that for the coldest (June) and the hottest (January) months, the Zona da Mata and Metropolitana regions had one of the highest temperature values and the highest humidity values for the state. In addition, these two mesoregions, Metropolitana and Zona da Mata, have the highest rainfall rates in Pernambuco state, with annual rainfall depths higher than 1200 and 900 mm, respectively [58].

In order to identify which regions of Pernambuco state would be most suitable for different purebred sheep, the critical THI limits per breed for environmental control—determined by McManus et al. [19] in an assessment of the distribution of sheep herds in Brazil and their relationship to climatic and environmental factors (Table 4)—were adopted.

**Figure 4:**
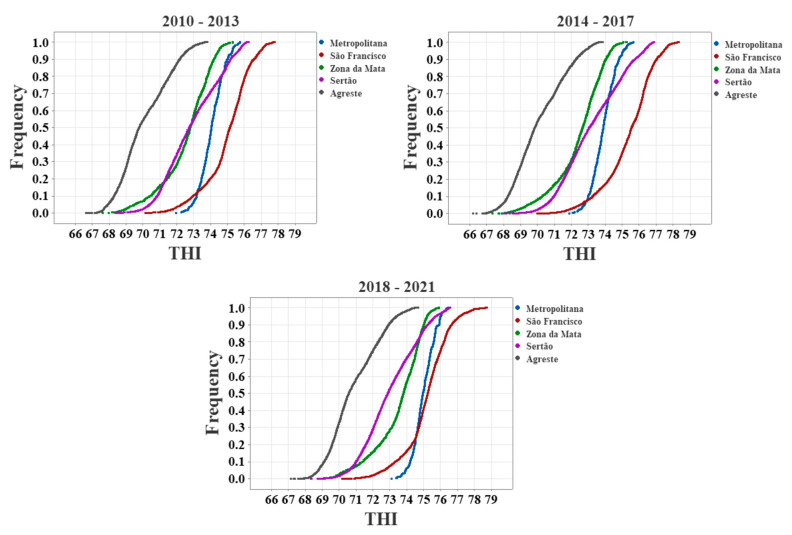
Cumulative distribution functions of the four-year THI values in Pernambuco state.

We also calculated the frequency distribution of the THI values of the presented mesoregions by their cumulative distribution functions, presented in quadrennials, as shown in Figure 4.

**Figure 5:**
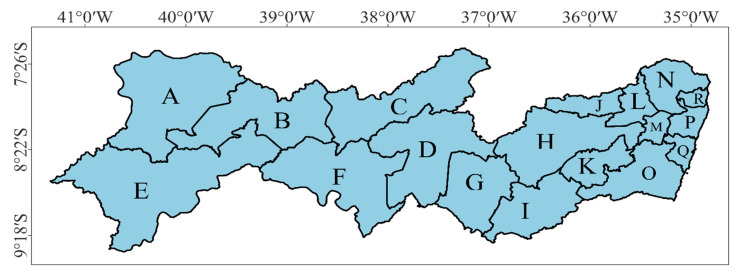
Map of Pernambuco state divided into its microregions. A—Araripina; B—Salgueiro; C—Pajeú; D—Moxotó; E—Petrolina; F—Itaparica; G—Vale do Ipanema; H—Vale do Ipojuca; I—Garanhuns; J—Alto Capibaribe; K—Brejo; L—Médio Capibaribe; M—Vitória de Santo Antão; N—Mata Setentrional; O—Mata Meridional; P—Recife; Q—Suape; R—Itamaracá.

According to Figure 3, there is high variation in THI values within the same mesoregion, especially in the larger ones such as the Sertão, São Francisco, and Agreste mesoregions. To enhance the discussion of the present study, Figure 5 shows Pernambuco state already divided into its microregions. The state is subdivided into nineteen microregions [64]; however, one of the microregions is Fernando de Noronha Archipelago, known for its high touristic value, which was not taken into account in the present study.

### 3.4. Hair x Wool Breeds

From Table 4, it can be seen that, except for the Bergamácia breed, the hair breeds have a higher tolerance to heat stress compared to wool breeds, based on THI values alone. This may be due to the fact that the hair structure protects the skin against direct solar radiation while promoting convection and heat loss through evaporation [65]. On the other hand, the wool cover makes water evaporation from the body more difficult, thus reducing heat loss through transpiration, although it also offers protection against direct solar radiation [66]. Therefore, the thermoregulatory process tends to occur more slowly in wool sheep [67]. However, it is not only the type of covering that influences the tolerance to heat stress of the animal. The adaptation to aggressive environments, with important physiological and structural changes, such as energy metabolism and body size, also determines the degree of adaptation of the sheep species to this type of stress [25,68].

According to the THI maps (see Figure 3), the hair breeds Rabo Largo, Morada Nova, Somali, Cariri, Santa Inês, and Dorper, as well as the wool breed Bergamácia, can be used in any mesoregion of the state. We can see that for the three periods used for cumulative distribution analysis, the mesoregions Agreste, Sertão, Zona da Mata, and Metropolitana presented 100% of THI values below 77, and São Francisco about 95%. From Figure 3, we can identify this region of THI > 77 as the westernmost portion of the Petrolina microregion, a territory where Santa Inês and Dorper sheep might be working close to their established THI limits. The White Dorper species has the lowest THI limit among hair sheep, so the extreme region mentioned might not be suitable for this breed.

The Rabo Largo sheep are known for their ability to walk long distances and to cope with harsh environmental conditions such as long periods of drought and high temperatures [69]. Although herds of this breed can be found in the São Francisco mesoregion [19], there is high potential for further exploitation of this breed in Pernambuco state, since it is usually chosen for the production of good quality meat which is closely related to the nutritional habits of indigenous human populations in other arid and semiarid parts of the world [70,71].

The Somali breed is adapted to a dry climate and scarce food supply and is restricted to the NEB. However, the population of Somali sheep mainly consists of small herds belonging to research institutes and a few herds belonging to breeders [72,73]. There is not much information about the Somali sheep in Brazil; most studies with this breed are based on crossbreeding to produce more meaty animals [74]. Furthermore, Bergamácia sheep have been considered robust, with lower maintenance requirements and easy handling; however, because of their smaller size, they are often considered to be less productive [17]. In the NEB, this breed was used mainly for intercurrent crossing with indigenous breeds [75]. Due to the replacement of local genetic groups by improved exotic breeds, the Bergamácia breed is practically no longer used by breeders [72].

McManus et al. [19] showed that wool breeds are mainly limited to the South and Southeast of Brazil (SEB), regions that have a more temperate climate and less aggressive environments. From Figure 3, it can be seen that the central area of the mesoregion Agreste (including the microregions of Garanhuns, Brejo, and the southern part of Ipojuca municipalities), as well as a small part of the extreme west of it, would be suitable for all wool breeds. It can be identified from Figure 4 that in the period from 2010 to 2017, the mesoregion of Agreste presented about 85% of THI values below 72, while for the 2018–2020 period, about 75%. From Figure 3, it is verified that the microregions of Vale do Ipanema and Médio Capibaribe presented THI > 72, extending to the region of Alto Capibaribe in the last quadrennium studied. These regions might not be suitable for Border Leicester, Lacaune, Merino, Corriedale, Ilê de France, Karakul, Creole, and Hampshire breeds. The eastern part of the mesoregion of Sertão (e.g., Pajeú and Moxotó) may also be suitable for the SAMM, Suffolk, Poll Dorset, East Friesian, and Texel breeds. However, this region is notorious for its extreme drought events [57,76], where less suitable animals may not properly develop.

The SAMM breed is an efficient feed converter and does extremely well in feedlot and pasture systems because of its ability to utilize low-quality roughage [77], presenting favorable growth and meat production attributes even under intensive rearing conditions [78]. It is one of the most common feedlot breeds throughout South Africa [79], being one of the most heat-adapted wool breeds, with potential for breeding in the recommended regions of Pernambuco state.

In addition to having low tolerance to heat stress, based on THI values (Table 4), the Border Leicester, Merino, Corriedale, Karakul, and Creole wool breeds are not recognized for being major producers of meat or milk [80]. For this reason, they were not considered feasible breeds for further exploitation in the state. McManus et al. [20] showed that, based on respiration rate, the Merino, Corriedale, and Creole breeds would usually be under stress in the semiarid region of Pernambuco.

### 3.5. Main Meat Production Breeds

The most popular breeds used for meat production include the Morada Nova, Santa Inês, Dorper, Suffolk, Poll Dorset, Texel, Ilê de France, and Hampshire breeds [81,82,83,84,85,86,87,88]. As mentioned, the Morada Nova, Santa Inês, and Dorper varieties can be raised in any mesoregion of the state, based on the THI maps (Figure 3). However, in the Petrolina microregion and most of the southwestern part of the Sertão mesoregion (south of the Araripina and southeast of the Salgueiro microregions), they may be at risk of being under heat stress on an average day, especially the Dorper sheep.

Morada Nova sheep represent one of the main sheep breeds in the NEB, since they are small in size and resistant to semiarid conditions, and this breed is a main source of protein for rural populations and small farmer holdings [89]. This breed uses a thermal storage mechanism to retain heat from the hottest times of the day, releasing it during the night or early morning, and they use panting as an immediate response to environmental heat stress, with sweating being a secondary response mechanism [90]. Santa Inês is the largest breed in the country, being reared in the Northeast, Midwest, and Southeast of the country [19,74], with Pernambuco state having one of the largest herds in Brazil [91]. It is the result of crossbreeding between Bergamácia, Morada Nova, and Somali, as well as other breeds, with no definitive standard, and is a dominant breed for meat production [92]. Based on the THI limit values (Table 2), the Santa Inês breed is not among the most heat resistant, in sixth place. However, Titto et al. [67] showed that after sun exposure, the increase in rectal temperature and respiration rate of Santa Inês sheep was significantly lower than the Morada Nova breed sheep, one of the most tolerant when taking into account only THI values.

Despite being an exotic breed in Brazil, Dorper sheep performed similarly to localized sheep (Morada Nova and Santa Inês) when exposed to heat stress [93]. In addition, Dorper sheep have coat characteristics that favor less thermal insulation and greater resistance to solar radiation [94]. Costa et al. [95] observed that in an environment exposed to direct radiation, Dorper sheep showed an increase in the area occupied by sebaceous glands in the dermis.

Temperate climate breeds such as Suffolk, Texel, and Ile de France show far worse physiological responses to heat stress compared to breeds adapted to the semiarid tropics such as Santa Inês and Morada Nova, representing extremely low heat tolerance [67,74,96]. From Figure 3, it can be seen that only the central region of the Agreste mesoregion (including the microregions of Garanhuns, Brejo, and the southern part of Ipojuca) and the small westernmost part of it are suitable for these breeds. However, because these breeds are less adapted to high temperatures, they may be living close to their established THI limit, as previously mentioned.

It is suggested that high-meat-production breeds are more likely to exhibit adverse effects of heat stress due to lower heat adaptation capacity as compared to resistant breeds that are more adapted to heat [88].

### 3.6. Main Milk Production Breeds

East Friesian and Lacaune are the two most common dairy breeds worldwide [97,98], both being wool breeds, with the East Friesian being slightly more heat resistant, based on THI values (Table 4). According to Figure 3, the central region of the Agreste mesoregion (including the microregions of Garanhuns, Brejo, and the southern part of Ipojuca), as well as a small part of the extreme west of it (northwest of the microregion of Vale do Ipanema), may be well suited to both of these breeds. The extreme north of the Pajeú microregion may also be suitable for East Friesian sheep.

### 3.7. Implications of the Study

The sheep industry in Brazil is growing, and research is needed to help breeders effectively increase investment in the sector. This study identifies the environmental conditions that are favorable for sheep production in Pernambuco, showing that breeds of interest that are not explored within the state can be inserted, allowing the local economy to take advantage of their good production levels, and also serving as a warning, and guiding producers so that breeding does not happen randomly, disrespecting the specific requirements of each sheep breed.

The association between the environment and production is dependent on technologies and investments, but these resources are not always available in Brazil, especially in the semiarid part of the NEB, and the absence of policy and guidance can negatively affect production, exposing the need for a management plan for sheep farming in Pernambuco that takes into consideration the environmental characteristics of the state, as it comprises a massive part of the entire Brazilian sheep herd.

Obviously, THI is not the only factor that limits the use of a breed in a given situation; however, comprehension of the spatial distribution of breeds, which is highly correlated with environmental control, can assist in the creation of environmental descriptors, classifying breeds for conservation. This is a first attempt to zone animal production by breed in Brazil. The development of a typology based on the methodology employed in this study can promote the creation of policies intended to increase the effectiveness of sheep farming, as the ability to adapt to climate change can determine whether producers, states, and countries will increase, maintain, or decrease their production levels or market share over the following years.

## 4. Conclusions

This study identifies the potential and thermal comfort conditions in different regions of Pernambuco state for different breeds of sheep found in Brazil, based on THI parameters and using geostatistical methods. We highlight that the THI values decreased from the Metropolitana mesoregion towards Agreste and increased towards Sertão and São Francisco.

Pernambuco has the proper conditions for the production of hair sheep, including three major meat-producing breeds (Morada Nova, Santa Inês, and Dorper), for almost all of its territory, except for the most southwestern part of the Petrolina microregion, a territory marked by high THI values during the entire studied period.

When it comes to wool species, the mesoregion of Agreste presents the greatest potential for breeding, especially the more adapted and better wool breeds for meat production such as Suffolk, Poll Dorset, and Texel, because almost the entire region showed THI values below 74. However, the less adapted wool breeds, such as Ilê de France and Hampshire, which are also good meat-production animals, may still face thermal stress in the microregions of Vale do Ipanema and Médio Capibaribe. The East Friesian sheep, one of the main dairy breeds, can also be well adapted in the mesoregion of Agreste, including the region known as the Dairy Basin of Pernambuco state.

## Figures and Tables

**Table 1:** Summary of descriptive statistics of annual THI values.

Year	Mean	^1^ Med	^2^ Min	^3^ Max	^4^ SD	^5^ CV	^6^ A	^7^ K
**2010**	72.45	72.56	65.94	76.97	2.25	3.10	−0.32	−0.79
**2011**	71.65	71.71	65.21	76.63	2.23	3.11	−0.21	−0.74
**2012**	72.84	72.89	65.85	77.86	2.50	3.43	−0.27	−0.73
**2013**	73.12	73.18	66.32	77.99	2.39	3.27	−0.27	−0.75
**2014**	72.18	72.26	65.39	76.90	2.39	3.31	−0.28	−0.79
**2015**	73.34	73.40	66.24	78.47	2.56	3.48	−0.23	−0.82
**2016**	73.50	73.61	66.42	78.30	2.53	3.45	−0.31	−0.84
**2017**	72.67	72.70	65.95	77.89	2.39	3.29	−0.23	−0.68
**2018**	73.05	73.20	67.02	77.05	1.93	2.64	−0.44	−0.60
**2019**	73.69	73.81	66.89	78.87	2.37	3.21	−0.24	−0.75
**2020**	72.64	72.65	66.43	77.10	2.13	2.93	−0.20	−0.91
**2021**	73.09	73.24	66.63	77.59	2.20	3.01	−0.33	−0.82

^1^ Median. ^2^ Minimum. ^3^ Maximum. ^4^ Standard deviation. ^5^ Coefficient of variation. ^6^ Asymmetry. ^7^ Kurtosis.

**Table 2:** Cross-validation for the tested geostatistical models.

Spherical					
Year	ME	RMSE	MSE	RMSSE	ASE
**2010**	−6.39 × 10^−5^	0.259214	−0.00048	0.7138	0.362777
**2011**	−5.08 × 10^−5^	0.257907	−0.00045	0.690961	0.372801
**2012**	−5.07 × 10^−5^	0.259716	−0.00046	0.694066	0.373771
**2013**	−5.26 × 10^−5^	0.260369	−0.00047	0.710843	0.36587
**2014**	−7.27 × 10^−5^	0.258842	−0.00049	0.711379	0.363453
**2015**	−5.34 × 10^−5^	0.261177	−0.00052	0.749804	0.34791
**2016**	−5.26 × 10^−5^	0.260877	−0.00047	0.69588	0.374477
**2017**	−4.15 × 10^−5^	2.59 × 10^−1^	−0.00045	0.704609	0.367699
**2018**	−2.75 × 10^−5^	0.260611	−0.00044	0.716682	0.363221
**2019**	−6.23 × 10^−5^	0.261536	−0.00045	0.703081	0.371541
**2020**	−7.11 × 10^−5^	0.260421	−0.00047	0.71442	0.364073
**2021**	−6.07 × 10^−5^	0.260687	−0.00046	0.701996	0.370905
**Gaussian**					
**Year**	**ME**	**RMSE**	**MSE**	**RMSSE**	**ASE**
**2010**	−0.00234	0.361035	−0.00638	0.944639	0.382196
**2011**	−0.00235	0.359701	−0.00639	0.934722	0.384795
**2012**	−0.00252	0.359492	−0.00695	0.950751	0.378098
**2013**	−0.00256	0.361166	−0.007	0.948248	0.380862
**2014**	−0.00234	0.360502	−0.0064	0.944673	0.381605
**2015**	−0.00255	0.36118	−0.00698	0.947964	0.380983
**2016**	−0.00254	0.362973	−0.0069	0.945865	0.383743
**2017**	−0.0024	0.361205	−0.00657	0.945479	0.382008
**2018**	−0.0025	0.364844	−0.0067	0.934608	0.39035
**2019**	−0.00229	0.363888	−0.00619	0.944569	0.385213
**2020**	−0.00212	0.366506	−0.00555	0.917904	0.399259
**2021**	−0.00229	0.365006	−0.00612	0.934848	0.390413
**Exponential**					
**Year**	**ME**	**RMSE**	**MSE**	**RMSSE**	**ASE**
**2010**	1.48 × 10^−4^	0.261364	0.00	0.585199	0.445757
**2011**	0.000167	0.260073	1.37 × 10^−5^	0.563429	0.460613
**2012**	0.000183	0.261855	2.46 × 10^−5^	0.568621	0.45959
**2013**	0.00019	0.26251	3.63 × 10^−5^	0.526857	0.497263
**2014**	0.000141	0.260972	−2.58 × 10^−5^	0.559033	0.4659
**2015**	0.000184	0.263107	2.39 × 10^−5^	0.555818	0.472411
**2016**	0.000184	0.263034	1.97 × 10^−5^	0.558827	0.469761
**2017**	0.000181	0.26155	2.33 × 10^−5^	0.604687	0.431644
**2018**	0.000208	0.262778	6.41 × 10^−5^	0.60554	0.43308
**2019**	0.000154	0.263722	3.33 × 10^−6^	0.572355	0.459804
**2020**	0.00012	0.262605	−4.41 × 10^−5^	0.52963	0.494776
**2021**	0.000158	0.262874	2.72 × 10^−6^	0.604837	0.433709

**Table 3:** Parameters for the fitted semivariogram models and Degree of Spatial Dependence.

Year	Model	Nugget Effect	Sill	Range	^1^ DSD
**2010**	Gaussian	0.1585	1.6217	59,902	9.77
**2011**	Gaussian	0.1316	1.7403	54,541	7.52
**2012**	Gaussian	0.1267	1.7982	57,355	7.00
**2013**	Gaussian	0.1287	1.7805	63,124	7.22
**2014**	Gaussian	0.1294	1.7170	55,485	7.51
**2015**	Gaussian	0.1287	1.8061	62,548	7.12
**2016**	Gaussian	0.1308	1.7594	61,548	7.39
**2017**	Gaussian	0.1296	1.7412	58,563	7.44
**2018**	Gaussian	0.1356	1.7220	62,551	7.87
**2019**	Gaussian	0.1318	1.7792	58,961	7.36
**2020**	Gaussian	0.1422	1.7163	60,512	8.27
**2021**	Gaussian	0.1356	1.7313	56,548	7.83

^1^ Degree of spatial dependence (%).

**Table 4:** Cover type and THI values for the main sheep breeds in Brazil [19] (adapted).

Breed	Cover Type	THI
Rabo Largo	Hair	81.93
Morada Nova	Hair	80.81
Somali	Hair	80.64
Bergamácia	Wool	79.40
Cariri	Hair	79.34
Santa Inês	Hair	78.27
Dorper	Hair	78.26
White Dorper	Hair	76.96
^1^ SAMM	Wool	75.92
Suffolk	Wool	74.61
Poll Dorset	Wool	74.55
East Friesian	Wool	74.55
Texel	Wool	73.67
Border Leicester	Wool	72.77
Lacaune	Wool	72.72
Merino	Wool	72.66
Corriedale	Wool	72.63
Ilê de France	Wool	72.63
Karakul	Wool	72.40
Crioula	Wool	72.10
Hampshire	Wool	71.99

^1^ South African Mutton Merino.

## Data Availability

Publicly available datasets were analyzed in this study. These data can be found here: https://climateengine.com.
